# G6PDH as a key immunometabolic and redox trigger in arthropods

**DOI:** 10.3389/fphys.2023.1287090

**Published:** 2023-11-17

**Authors:** Bruno Moraes, Renato Martins, Cintia Lopes, Ronald Martins, Angélica Arcanjo, Jhenifer Nascimento, Satoru Konnai, Itabajara da Silva Vaz, Carlos Logullo

**Affiliations:** ^1^ Instituto de Bioquímica Médica Leopoldo de Meis, Universidade Federal do Rio de Janeiro, Rio de Janeiro, Brazil; ^2^ Instituto Nacional de Ciência e Tecnologia em Entomologia Molecular–INCT, Rio de Janeiro, Brazil; ^3^ Programa de Computação Científica, Instituto Oswaldo Cruz, IOC, Rio de Janeiro, Brazil; ^4^ Laboratory of Infectious Diseases, Hokkaido University, Sapporo, Japan; ^5^ Centro de Biotecnologia and Faculdade de Veterinária, Universidade Federal do Rio Grande do Sul (UFRGS), Porto Alegre, Brazil

**Keywords:** glucose-6-phosphate dehydrogenase, redox, immunometabolism, NADPH, glucose metabolism, arthropods, pentose phosphate pathway

## Abstract

The enzyme glucose-6-phosphate dehydrogenase (G6PDH) plays crucial roles in glucose homeostasis and the pentose phosphate pathway (PPP), being also involved in redox metabolism. The PPP is an important metabolic pathway that produces ribose and nicotinamide adenine dinucleotide phosphate (NADPH), which are essential for several physiologic and biochemical processes, such as the synthesis of fatty acids and nucleic acids. As a rate-limiting step in PPP, G6PDH is a highly conserved enzyme and its deficiency can lead to severe consequences for the organism, in particular for cell growth. Insufficient G6PDH activity can lead to cell growth arrest, impaired embryonic development, as well as a reduction in insulin sensitivity, inflammation, diabetes, and hypertension. While research on G6PDH and PPP has historically focused on mammalian models, particularly human disorders, recent studies have shed light on the regulation of this enzyme in arthropods, where new functions were discovered. This review will discuss the role of arthropod G6PDH in regulating redox homeostasis and immunometabolism and explore potential avenues for further research on this enzyme in various metabolic adaptations.

## 1 Introduction

Glucose homeostasis in the circulatory system, which is vital for meeting the energy demands of organisms, is regulated by hormones, and relies on the balance between glucose uptake, synthesis, and oxidation (Nelson and Cox, 2019). However, glucose metabolism not only serves as an energy source but also plays a crucial role in preventing oxidative damage from reactive oxygen species. The pentose phosphate pathway (PPP) links glucose metabolism to redox homeostasis (Kruger and von Schaewen, 2003). The PPP begins with glucose-6-phosphate (G6P), the first product of glycolysis produced by hexokinase. The pathway comprises two branches: the oxidative phase, which generates ribulose-5-phosphate and reduces NADP + to NADPH, and the non-oxidative phase, which contributes to the synthesis of 5-carbon sugars. Ribulose-5-phosphate can reversibly isomerize to ribose-5-phosphate, which is involved in several essential metabolic pathways, such as the synthesis of nucleotides and nucleic acids (ribose-5-phosphate) and the production of aromatic amino acids via the erythrose-4-phosphate pathway. In most organisms studied to date, these processes predominantly occur in the cytosol or in plastids of plants (Kruger and von Schaewen, 2003; Keller et al., 2014).

In humans, PPP has been recognized as a crucial pathway strongly associated with several important pathologies. For example, exposure to pro-oxidant agents can induce hemolytic anemia, particularly in individuals consuming fava beans. Additionally, a deficiency in reduced glutathione (GSH), which can be caused by antimalarial agents and sulfa antibiotics, has been found to positively correlate with PPP disorders (Cordes, 1926). On the other hand, PPP enzymes from non-mammalian organisms such as arthropods have received little attention so far in the medical field.

In *Bombyx mori*, it has been observed that G6PDH activity remains consistently high during both diapause and embryonic development but experiences a rapid decline just before hatching (Suzuki and Miya, 1975). The maintenance of the diapause state is essential for the organism’s survival during unfavorable periods, and G6PDH plays a pivotal role in facilitating the metabolic adaptations that occur throughout this process (Suzuki and Miya, 1975).

In the case of the tick *Rhipicephalus microplus*, the presence of G6PDH-A and G6PDH-C genes in fed female ticks serves a crucial function in enhancing their resistance to oxidative stress, which occurs during the feeding process. Additionally, the level of transcript expression in fed female ticks may be contingent upon variables such as the volume of the blood meal ingested and the duration of time adult females spend on the host in comparison to adult males (Olafson et la., 2011). Moreover, the G6PDH is linked to the remodeling of energy metabolism in these tick cells exposed to oxidative stress (Della Noce et al., 2019; Della Noce et al., 2022). In the mosquito *A. aegypti* it was previously observed high levels of glucose 6-phosphate dehydrogenase (G6PDH) activity at the very beginning of *Aedes aegypti* embryogenesis, which nevertheless decreased up to 5 HAE, indicating its significant involvement in the initial stages of embryonic development, prior to the retraction of the germ band (Vital et al., 2010).

Clearly, G6PDH has been extensively studied in mammals; however, there are some limited researches been conducted on the role of this enzyme in arthropods, leaving major gaps of knowledge on its function in oxidative homeostasis and physiology. This review aims to address this gap by focusing on arthropod G6PDH and its role in the regulation of pentose phosphate pathway, oxidative homeostasis, immunometabolism control, and other metabolic pathways. Furthermore, this review will discuss potential avenues for research to shed light on the important roles of this enzyme in a range of metabolic adaptations in arthropods.

## 2 Glucose metabolism

**FIGURE 1:**
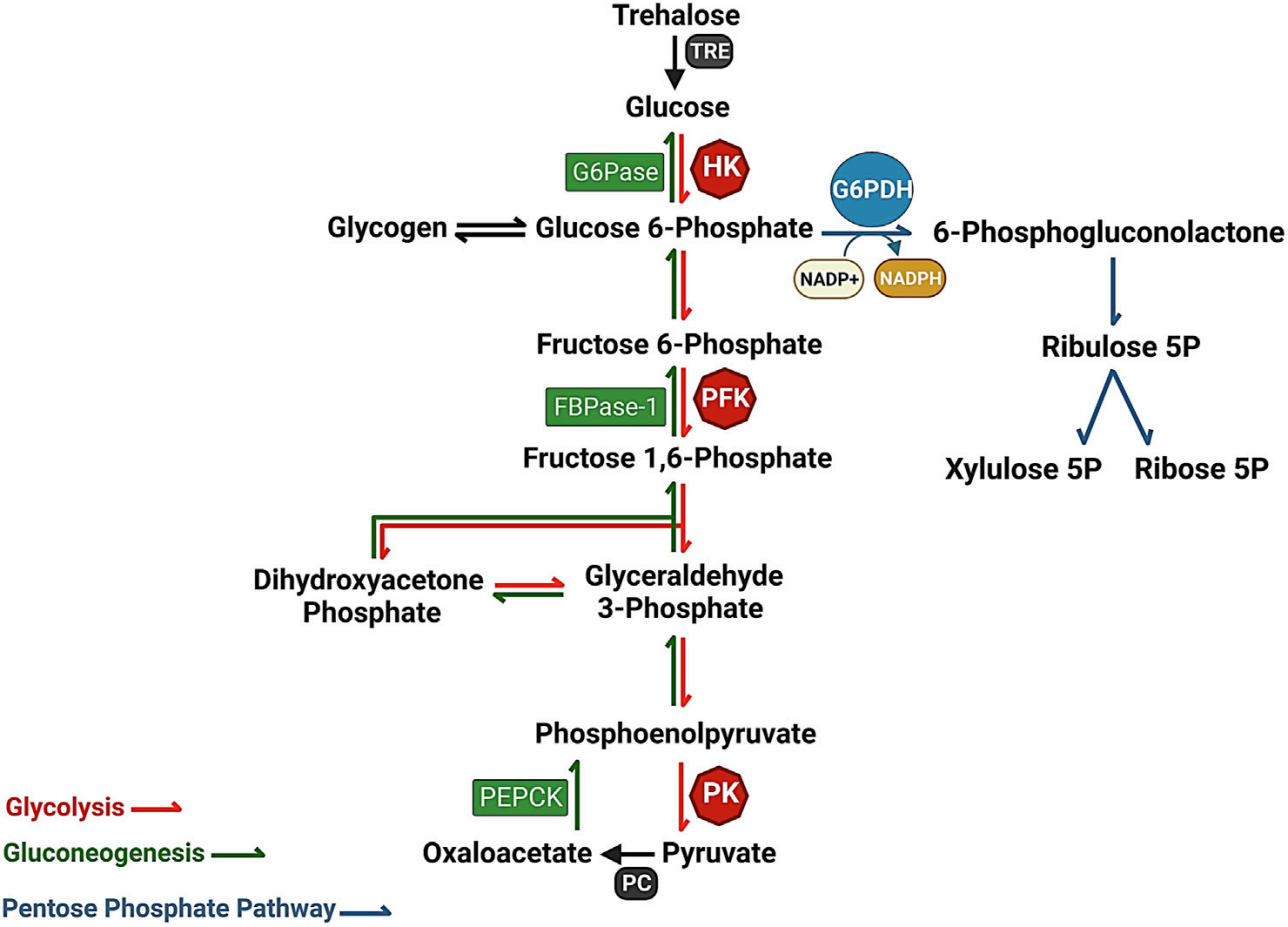
The interaction of metabolic pathways, Glycolysis (red), Pentose phosphate pathway (gray), and gluconeogenesis (green), providing a constant flow of energy and substrates for cellular metabolism. Glucose-6-phosphate dehydrogenase (G6PDH) serves as a key catalyst in the Pentose Phosphate Pathway, driving the rate-limiting step. G6PDH converts glucose-6-phosphate to 6-phosphogluconolactone, facilitating the production of NADPH, an essential cofactor involved in antioxidant defense and macromolecule synthesis.

Glucose can be directed into various metabolic pathways depending on cellular needs. It can be utilized for the synthesis of structural polymers such as extracellular matrix and cell wall polysaccharides, stored as glycogen, oxidized through the pentose-phosphate pathway to generate ribulose-5-phosphate, or undergo glycolysis to produce pyruvate. The liver plays a pivotal role in maintaining glucose homeostasis by regulating several metabolic pathways related to glucose, including glycogenesis, glycogenolysis, glycolysis, and gluconeogenesis (Han et al., 2016). In mammals, the balance between glucose uptake and storage through glycogen synthesis as well as its release via glycolysis or *de novo* glucose synthesis via non glycemic source (amino acid, glycerol and lactate) is crucial for maintaining blood glucose levels (Figure 1).

In insects, trehalose (synthesized in the fat body using glucose as a precursor) is considered the major sugar that maintains circulatory homeostasis. Trehalose serves as the primary sugar in the hemolymph and plays important roles in insect reproduction, response to environmental stress, and overall metabolism. It acts as a carbon source for various metabolic pathways, including the pentose phosphate pathway (PPP), gluconeogenesis, and glycolysis (Figure 1) (Murphy and Wyatt, 1965; Crowe et al., 1984; Elbein et al., 2003; Chang et al., 2022). Trehalase, the glycosidase enzyme responsible for trehalose hydrolysis into two glucose molecules, is an essential enzyme found in diverse organisms such as bacteria, algae, plants, and insects (Hehre et al., 1982; Myrbäck and Örtenblad, 1937; Kalf and Rieder, 1958; Mori et al., 2009) (Figure 1). This protein plays a distinct and significant role in sugar homeostasis (Avonce et al., 2006). The mosquito *A. stephensi* shows a constitutive expression of trehalase during larvae aquatic development and in mosquito’s tissues which exhibit an upregulation until 42 h starvation. Trehalase seems to have an important role in reproduction of *A. stephensi*, after blood meal we can see the gene is upregulated in the fat body and ovary 24 and 48 h after blood meal respectively suggesting an interesting role in the insect reproduction. Surprisingly this gene may have a role in the parasite development and survival in *Plasmodium vivax* infected mosquitos which shows an upregulated expression in the midgut and salivary gland suggesting an interestingly relation between parasite-host metabolism (Tevatiya et al., 2020).

During glycolysis, a portion of the energy present in the glucose molecule is stored as ATP and NADH, while the majority remains in the pyruvate product. The conversion of six-carbon glucose into two three-carbon pyruvate molecules takes place via ten individual steps. On the other hand, Gluconeogenesis is a metabolic process that involves the synthesis of glucose from non-glucose precursor molecules, such as lactate, pyruvate, and oxaloacetate, among others. The process involves seven reversible reactions that are catalyzed by enzymes shared with glycolysis. Glycolysis and gluconeogenesis are central metabolic pathways that are regulated by specific enzymes in opposite directions to maintain the correct direction of the net flux (Hers and Hue, 1983; Nelson and Cox, 2019).

Glucose also can be oxidized through the pentose phosphate pathway, which is a metabolic process with two main functions: producing NADPH, a reducing agent used in biosynthesis, and producing ribose-5-phosphate, a sugar used in nucleic acid synthesis (Horecker, 2002; Nelson and Cox, 2019). The first step in this pathway is catalyzed by glucose-6-phosphate dehydrogenase (G6PDH), which converts glucose-6-phosphate to 6-phosphogluconolactone and produces an NADPH molecule. G6PDH’s regulation and physiological roles were first described in 1931 (Kornberg et al., 1955). Deficiency in G6PDH can lead to hemolytic anemia, a condition where red blood cells are destroyed due to the inability to neutralize free radicals, resulting in jaundice and fatigue (Luzzatto et al., 2016; Martini and Ursini, 1996; Notaro et al., 2000). Moreover, G6PDH plays a crucial role in cellular protection against oxidative stress by maintaining redox balance within the cell (Nelson and Cox, 2019; Kletzien et al., 1994; Stanton, 2012).

## 3 G6PDH and redox homeostasis

**FIGURE 2:**
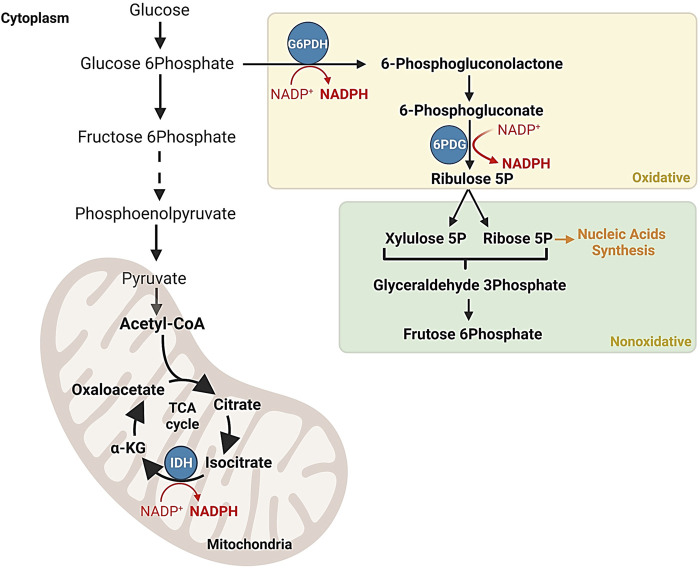
NADPH production in the pentose phosphate pathway and the Krebs cycle. Schematic representation of regulatory mechanisms ensuring the efficient synthesis of NADPH, a vital cofactor involved in antioxidant processes and macromolecule synthesis. The Krebs cycle indirectly contributes to NADPH production through reactions such as the isocitrate dehydrogenation to *α*-ketoglutarate by the isocitrate dehydrogenase enzyme complex.

G6PDH is a crucial enzyme in the pentose phosphate pathway (PPP), which was first partially elucidated by Nobel laureate Otto Warburg in the 1930s. The PPP has been extensively studied in various organisms, and in most cases, the enzymes involved are in the cytoplasm, where they generate essential metabolites and redox cofactors. In mammals, PPP occurs in the cytoplasm of cells in various organs, including the liver, mammary glands, and adrenal glands, where it plays a vital role in generating high levels of NADPH required for biosynthetic processes (Figure 2) (Warburg and Christian, 1931; Baldwin and Krebs, 1981; Couri and Racker, 1959; Dickens and Williamson, 1956; Frederiks et al., 2007; Horecker, 2002; Jin et al., 2018; Lewis et al., 2014).

Since PPP initial discovery, extensive research has been conducted on G6PDH in various organisms, revealing its diverse roles in metabolism (Chefurka, 1957; Kirkman, 1962; Kuby and Noltmann, 1966; Horie, 1967; Olive and Levy, 1967). G6PDH, a classical oxidoreductase that relies on NADP+, is the most studied enzyme in the PPP and is considered the rate-limiting step in its oxidative branch (Kletzien et al., 1994). It catalyzes the oxidation of glucose-6-phosphate and the reduction of NAD phosphate (NADP) to produce 6-phosphogluconate and NADPH (Berg et al., 2002). The significance of G6PDH extends beyond carbohydrate metabolism and to various biochemical processes. Together with 6-phosphogluconate dehydrogenase (6PGD), G6PDH is involved in the initial steps of the oxidative pathway known as the hexose monophosphate (HMP) pathway. The discovery of these enzymes played a pivotal role in uncovering the importance of coenzyme NADP (Berg et al., 2002) (Figure 2).

G6PDH deficiency is the most common gene mutation worldwide; it affects nearly 400 million people and has been classified by the World Health Organization (Vulliamy et al., 1998; Minucci et al., 2009; Li et al., 2015b; Ouattara et al., 2016; Gunawardena et al., 2017). The first description of G6PDH mutation dates to the 19th century when children who ate fava beans showed symptoms of anemia and hemoglobinuria, known as favism. Later in the 1920s, the side effects of aminoquinolines were described (Fermi and Martinetti, 1905; Cordes, 1926). Currently, G6PDH deficiency can be classified according to the residual activity of the enzyme, ranging from the most severe form (Class I) to the least severe form (Class V) (Gómez-Manzo et al., 2014; Gómez-Manzo et al., 2015). Interestingly, G6PDH mutations appear to be more prevalent in regions where malaria is endemic, raising the possibility of a protective effect against this disease (Gunawardena et al., 2017; Mbanefo et al., 2017).

G6PDH is considered an important housekeeping marker, playing an essential role in producing NADPH, an electron donor acting as a cofactor in several metabolic pathways, including fatty acid synthesis, cholesterol, and steroid hormone biosynthesis, and DNA precursor formation (Berg et al., 2002; Cao et al., 2020). G6PDH has a broad tissue expression and activity levels in different organisms, making it a critical enzyme in maintaining redox homeostasis in living cells (Luzzatto and Battistuzzi, 1985; Vulliamy et al., 1992). Among different cell types, red blood cells (RBCs) have a strong dependence on G6PDH due to their lack of mitochondria, making G6PDH an essential NADPH source for preventing RBCs against oxidative damage caused by reactive oxygen species (ROS) (Prankerd, 1961; Manganelli et al., 2010). G6PDH mutations in RBCs can reduce their tolerance to oxidative stress, leading to a decreased amount of NADPH and reduced glutathione (GSH) regeneration by glutathione reductase (GR) reaction. This can lead to ROS accumulation, causing RBC hemolysis and reduced RBC clearance (Beutler, 1978; Efferth et al., 2006). The findings corroborate a positive correlation between the prevalence of G6PDH mutations and malaria endemism, suggesting a potential protective role against malaria (Gunawardena et al., 2017; Mbanefo et al., 2017).

As a result of endo crossing of two *B. mori* strains, the F1 has outperformed their parents in several parameters such as body weight, silk gland and cocoon weight. Xiao et al., 2020, using multi-omics strategies like transcriptome and proteome, showed that the gene BGIBMGA012872 was upregulated in hybrid populations. This gene encodes G6PDH, suggesting an improved capacity to deal with environmental and pro-oxidant factors, besides a putative upregulated G6PDH activity, may increase NADPH levels, contributing not only to redox homeostasis but also the biosynthesis of new biomolecules.

G6PDH is classically regulated by NADPH/NADP + ratio (Holten et al., 1976). Recently some studies are showing some new and important mechanisms to regulate this enzyme. Some post-translational modifications have been showing as a key regulator of G6PDH. G6PDH can be phosphorylated in serine and tyrosine residues, including S40 and Y112. When S40 is phosphorylated promotes an improved immune response which is related with T cell metabolism via G6PDH-NADPH redox system (Gu et al., 2021). Y112 phosphorylation increases G6PDH activity and consequently PPP metabolites production like NADPH and ribose-5-phosphate (Ma et al., 2021). In the last years epigenetic studies showed relation with G6PDH activity, inhibition of histone deacetylase promotes G6PDH expression which can be related to higher activity (Makarona et al., 2014), in the other hand methylation of histone residues can inhibit G6PDH expression (Lu et al., 2022).

G6PDH is essential for proper embryonic development and a complete absence of its activity can be lethal due to the high sensitivity of embryos to oxidative stress. In mice, a full ablation of G6PDH activity prevented embryonic development beyond a certain stage, likely due to the inability to deal with blood oxygen exposure. Similarly, zebrafish embryos with G6PDH knockdown displayed impaired development, which was rescued by co-injection of human G6PDH cRNA. These findings were also observed in *Caenorhabditis elegans*, which showed severe defects in hatching, membrane-associated abnormalities, including enhanced permeability, abnormal lipid composition, defective polarity, and cytokinesis (Longo et al., 2002; Chen et al., 2018; Wu et al., 2018).

G6PDH’s role in maintaining oxidative homeostasis has been observed in arthropods. Studies on *R. microplus* ticks have demonstrated that G6PDH plays a vital role in oxidant detoxification; it shifts glucose to the PPP pathway and increases its transcription and activity to produce NADPH, which acts as an antioxidant agent contributing to redox homeostasis (Della Noce et al., 2019). Moreover, the knockdown of G6PDH in *R. microplus* tick embryonic cell line (BME26) induced oxidative stress, while NADP-ICDH provided NADPH in a compensatory mechanism (Della Noce et al., 2019). It was suggested that G6PDH activity ensures the high tolerance to hydrogen peroxide observed BME26, and this was supported by the induction of gluconeogenesis as a compensation strategy that involved metabolic enzymes such as NADP-ICDH, G6PDH, and PEPCK (Della Noce et al., 2022). This phenomenon resulted in glycogen accumulation and glucose uptake, which supported the pentose phosphate pathway to maintain NADPH synthesis, leading to cell survival and growth. It is important to note that other enzymes may also participate in the anti-oxidative response in glycolytic cells. Sodium nitroprusside (SNP) is a well-known inductor of oxidative stress. When *D. melanogaster* larvae were exposed to this compound, G6PDH and other antioxidant enzymes like superoxide dismutase, thioredoxin reductase and glutathione-S-transferase showed a higher activity compared to the control, suggesting an integrated mechanism to achieve redox homeostasis (Lozinsky et al., 2012; Lozinsky et al., 2013).

G6PDH activity is also important to protect against heavy metal pollution: acute or persistent contamination with heavy metal induces several metabolic responses and ultrastructural changes, activating the antioxidant defense system (Atli and Canli, 2010; Li et al., 2015a; Zhou et al., 2016; Zhou et al., 2017; Zhou et al., 2017; Das et al., 2019). NADPH is essential to maintain the GSH/GSSG ratio in *Sinopotamon henanense* hepatopancreas. Acute exposure to Cadmium Cd2+ lead to a reduction in PPP pathway activity resulting in less available NADPH; on the other hand, sub-chronic doses of this metal induced a higher G6PDH activity and NADPH content, suggesting that the enzyme may serve as a biological marker of contamination. In another example, the water flea *Daphnia magna* showed an increased G6PDH activity after mercury exposure (Tian et al., 1999; De Coen et al., 2001; Winzer et al., 2002; Pierron et al., 2007). Being able to establish a relationship between environmental conditions and metabolism can lead to a better understanding of adaptation landmarks in each species. Antioxidant enzymes are extremely sensitive to stress conditions like contamination, and consistent knowledge about these enzymes is a powerful tool to monitor habitats (Berra et al., 2004; Niemi and McDonald, 2004; Bonada et al., 2006).

**FIGURE 3:**
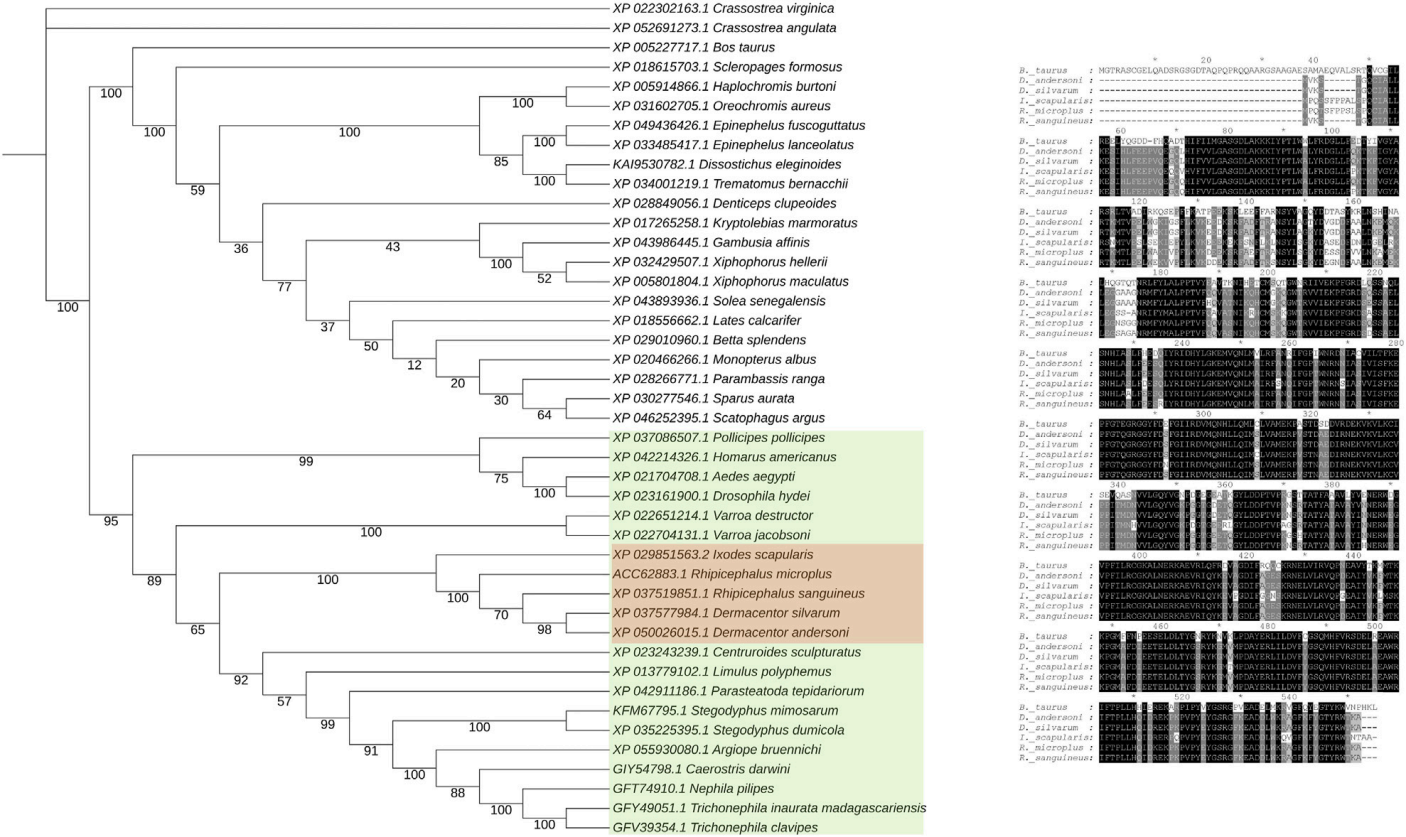
Phylogenetic analysis of glucose-6-phosphate dehydrogenase amino acid sequences. A BLASTP search was performed against the non-redundant database to identify relevant sequences. For each species, a single representative sequence was selected based on identity criteria. Subsequently, these sequences were aligned using the MAFFT online tool (Rozewicki et al., 2019), ensuring proper alignment for accurate phylogenetic analysis. The phylogenetic tree was constructed using the RAxML-HPC2 tool (Stamatakis, 2014) available on the CIPRES Science Gateway, encompassing 1,000 Bootstrap iterations. This tool employs maximum likelihood methods to infer phylogenetic relationships. The tree visualization was created using the iTOL (Interactive Tree Of Life) platform (Letunic and Peer, 2021), enhancing clarity and facilitating the interpretation of the tree’s branching patterns. Bootstrap values are shown on each node.

The unique parameters of these enzymes may be used as a fingerprint of each specific species present in the ecosystem. Tick G6PDH appear to have similar evolutionary origins (Figure 3), different ticks sharing the same branch in the phylogenetic tree. It is possible to see two distinct branches containing invertebrates and vertebrates. Analyzing 4 different species of *Plecoptera* insects, *P. marginata* showed a reduced G6PDH activity when compared to other *Plecoptera.* On the other hand, *P. marginata* exhibited the highest antioxidant capacity sum, which can indicate the important role of other NADPH- generating enzymes such as isocitrate dehydrogenase and malic enzyme (Sanz et al., 2010).

**FIGURE 4:**
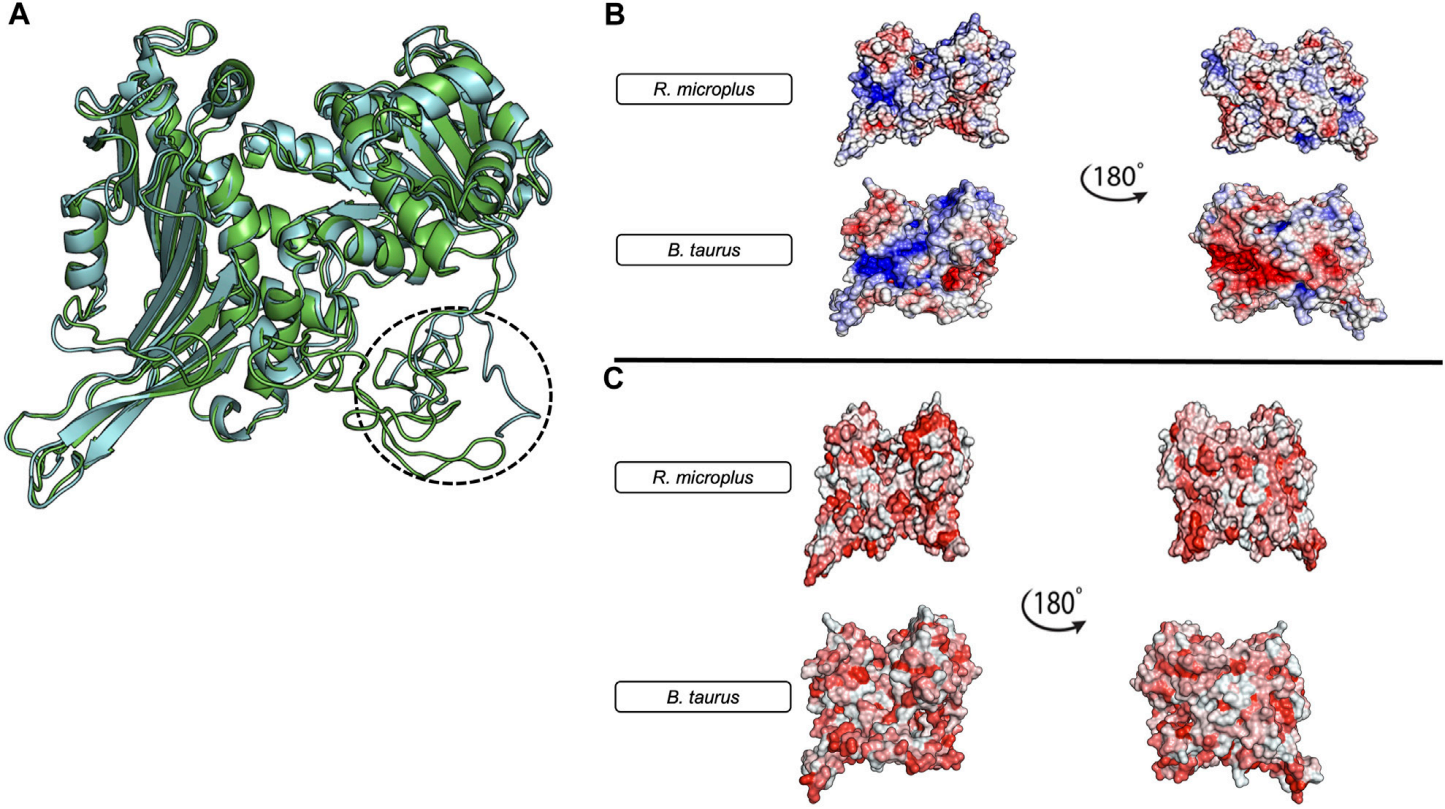
Structural Comparison and Physicochemical Properties of *Bos taurus* and *Rhipicephalus microplus* G6PDH models. **(A)** *R. microplus* G6PDH (green) and *B.taurus* G6PDH (Gray). The three-dimensional models were constructed using the I-TASSER server (Zhang, 2008) and their energies were minimized using the ReFold platform (McGuffin et al., 2018). The *Bos taurus* model was obtained from GenBank (Benson et al., 2013) GenBank Accession: XP_005227717.1, Isoform 3). **(B)** The electrostatic profiles of both protein models, with 180° rotations shown on the right. The red areas represent negative charges, while blue areas indicate positive charges. **(C)** The hydrophobicity distribution in the protein models, with 180° rotations also presented on the right. Red regions signify increased hydrophobicity. All figures were generated using the PyMol software (DeLano, 2002).

At the protein structure level (Figure 4), tick G6PDH and mammalian G6PDH show different regions, and it is possible to observe a unique N-terminus in the tick protein (Figure 4A). These proteins also show some other important differences including electrostatic features near the active site, where it is possible to notice more positive areas in the bovine protein when compared to the tick. These differences also encompass the active site, potentially creating a totally different electrostatic environment in the bovine protein (Figure 4B). The hydrophobic protein surface also shows some interesting differences, especially in the N-terminal region, and a hydrophobic hinge near the active site in tick G6PDH (Figure 4C). These differences, combined with the specific electrostatic surfaces, can be used as possible targets for drug development or defining the new immune protection strategies against arthropods that transmitting diseases.

## 4 Immunometabolic aspects of pentose phosphate pathway

Arthropods engage with a diverse array of microorganisms and possess intrinsic immunity that can decrease the likelihood of infection, or combat it more effectively (Pennacchio and Strand, 2006; Wang et al., 2018). The innate immune response encompasses various defense mechanisms, such as humoral responses, redox metabolism, physical and chemical barriers, and recruitment of hemocytes (Baxter et al., 2017; Fogaça et al., 2021). However, the activation of immunological mechanisms requires signaling molecules, synthesis of antimicrobial peptides, and proliferation of hemocytes, resulting in an energy expenditure for the host (Bajgar et al., 2015; Samaddar et al., 2020). Consequently, achieving a balance between energy cost and immune response is vital to mount an efficient defense without causing significant harm to the host. This equilibrium between metabolism and immunity is referred to as immunometabolism (Samaddar et al., 2020; O'Neill et al., 2016).

The immune response can be influenced by the availability of glucose. In mammals, immune cell activation is associated with elevated glycolysis and decreased mitochondrial metabolism, resulting in a reliance on high levels of glucose (Dolezal et al., 2019). In insects, transcriptome analysis of activated hemocytes revealed upregulation of genes encoding proteins involved in glycolytic functions (Bartholomay et al., 2004; Choi et al., 2012). Additionally, during the proliferation and differentiation of hemocytes in *D. melanogaster* infected with a parasitoid wasp, there was an increase in glucose consumption and lactate production. This suggests that activated immune cells in insects can adapt their metabolic response (Bajgar et al., 2015).

Furthermore, arthropods employ an additional defense mechanism by generating reactive oxygen species (ROS) as a protection against pathogens. Increased ROS levels can enhance pathogen elimination and safeguard the host from fungi, bacteria, or parasites. Plasmocyte cells in the tick *R* **.** *microplus* exhibited ROS production when exposed to bacteria, zymosan, or phorbol 12-myristate 13-acetate. In mosquitoes, higher systemic ROS levels have been associated with improved survival against bacterial infections (Pereira et al., 2001; Molina-Cruz et al., 2008). On the other hand, certain parasites like trypanosomatids exploit elevated ROS levels and utilize them as a signal for proliferation (Kakani et al., 2019; Maldonado et al., 2020). In *Drosophila melanogaster* gut, nicotinamide adenine dinucleotide phosphate oxidase (DUOX) plays a key role in antimicrobial activity. NADPH oxidases facilitate the transfer of electrons from NADPH to molecular oxygen, leading to the generation of the free radical superoxide and various downstream reactive oxygen species (ROS). The activation of NADPH oxidases, which results in ROS production, is involved in a wide array of functions, including host defense, cellular signaling, gene expression regulation, cell differentiation, metabolic processes, post-translational protein modifications, stress responses, and the maintenance of tissue homeostasis. (Bedard and Krause, 2007; Sorce and Krause, 2009). Many cells express several NOX isoforms; differences in subcellular distributions and activation mechanisms of different NOX isoforms might explain the non-redundancy in their functions (Bedard and Krause, 2007; Sorce and Krause, 2009; Gao et al., 2012). Dual oxidase enzymes, including DUOX, generate ROS, superoxide, or hydrogen peroxide by utilizing oxygen and NADPH as an electron donor (Donkó et al., 2005; Ha et al., 2005; Champion and Xu, 2017).

In this context, the involvement of enzymes and pathways that reduce NADP+ is of great significance, notably the pentose phosphate pathway, which serves to convert NADP to NADPH. Accordingly, a study conducted on *Culex pipiens* mosquitoes revealed that ingestion of *Plasmodium relictum*-infected blood triggers an increase in the transcription of NOS and G6PDH, providing evidence for the involvement of PPP in this immunological control process (Delhaye et al., 2016). In the non-oxidative branch of PPP, ribose 5-phosphate is produced. Recent research has indicated that the extracellular nucleoside adenosine can regulate energy allocation during bacterial infections in *D. melanogaster*. This signal plays a significant role in host immunity, influencing metabolism and the host-pathogen relationship (Bajgar et al., 2015; Bajgar and Dolezal, 2018).

Another important NADPH function as an essential enzymatic cofactor. Important enzymes as glutathione/glutathione disulfide (GSH/GSSG) and thioredoxin (Trx) are NADPH dependent (Arner, 2009). To regenerate then from to oxidate state to reduced state two enzymes are required thioredoxin reductase (TrxR) and glutathione reductase (GR) (Halliwell, 1999) which has the function to transfer electrons from NADPH to both. In mammals this system is completely separated, in the other hand some invertebrate such as Flatworm and *Schistosoma mansoni* has encoded only one single gene, thioredoxin glutathione reductase (TGR) (Alger and Wiliams, 2002; Salinas et al., 2004; Otero et al., 2010). In the last 10 years this enzyme complex has received more attention, Ross et al., 2012; Petukhova et al., 2023 showed that thioredoxin glutathione reductase enzyme can be a potential target against these invertebrate animals.

NADPH is a very important immune cofactor. Since DUOX is a NADPH oxidase involved in immune protection in several arthropods’ species like *Diptera; Lepidoptera; Hemiptera; Coleoptera and Hymenoptera* (Ha et al., 2005; Molina-Cruz et al., 2008; Kumar et al., 2010; Azzouz-Olden et al., 2018; Lin et al., 2020; Zeng et al., 2022). *Drosophila melanogaster* DUOX reduced activity leads to modification commensal community gut members (Kim and Lee, 2014). Silencing of this enzyme results in a higher mortality when fed with bacteria but not with sterile food (Ha et al., 2005). Results with *Anopheles gambie* also suggest DUOX role in immunometabolism in insects (Kumar et al., 2010). Interestingly this seems not to have the same role in ticks. Silenced ticks show more resistance to *Borrelia burgdorferi* infection due activation of specific tick innate immune pathway genes (Yang et al., 2014). Nitric oxide (NO) is a very well known as second-messenger with many different functions and targets (Khan and Hare, 2003). Nitric oxide synthase (NOS) is the responsible enzyme to produce NO using as substrate the amino acid arginine in presence of NADPH (Snyder and Bredt, 1992; Davies, 2000). NO displays many different roles in invertebrates, vasodilator for blood-sucking arthropods, has central importance in primitive olfactory like system and melanine production (Ribeiro and Nussenzveig, 1993; Colasanti et al., 1997; Palumbo et al., 1997). In mammalian endothelial cells, G6PDH activity showed important function to produce NADPH to sustain REDOX metabolism controlled and it is related with bioavailable NO, furthermore overexpression of G6PDH in these cells showed decreased ROS accumulation and increased amount of bioavailable NO^·^ (Leopold et al., 2003).

**FIGURE 5:**
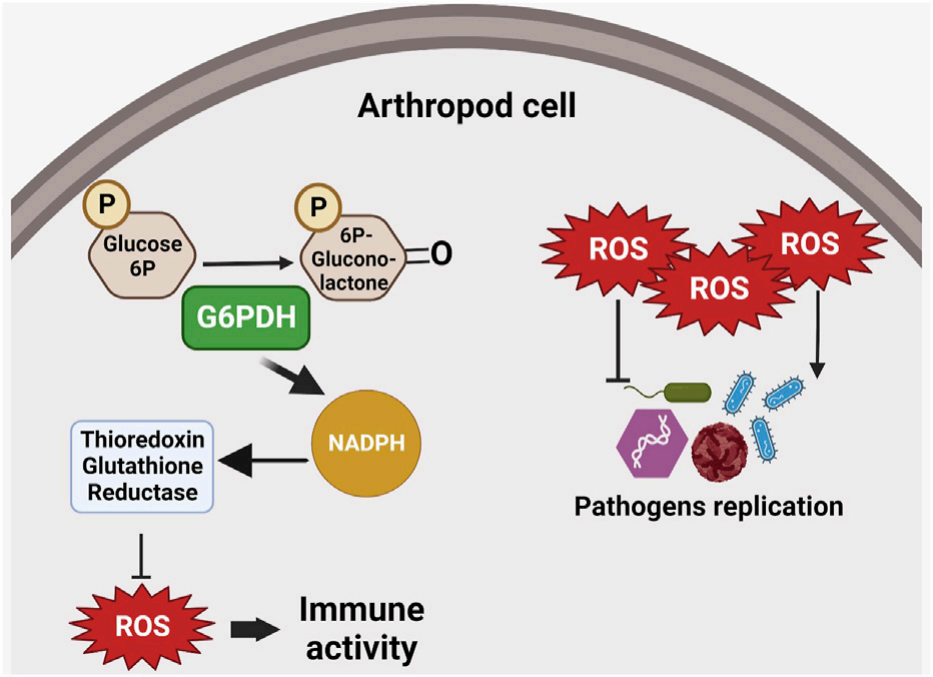
G6PDH role in immune responses. G6PDH enzymatic activity may increase NADPH levels and thus interfere with intracellular redox balance. Reactive oxygen species (ROS) can interfere with pathogen replication, suggesting an indirect role for G6PDH in the host’s immune response. ROS can inhibit replication in certain pathogens (e.g., bacteria and fungi), or induce parasite replication.

During virus infection in arthropods, G6PDH can play a relevant role. The White Spot Syndrome Virus (WSSV) (genus Whispovirus, family Nimaviridae) is a dsDNA virus which can kill almost 100% of infected shrimps in 10 days (van Hulten et al., 2001; Yang et al., 2001). During infection, the shrimp metabolism changes to a Warburg effect condition, increasing glucose transport and several glycolytic enzymes (Chen et al., 2011; Huang et al., 2015; Chen et al., 2016; Hernández-Palomares et al., 2018). Th ese infection-triggered changes are also reflected in PPP enzymes such as G6PDH, which shows higher activity during infection, while the enzyme substrate is consumed (Chen et al., 2016). While G6PDH importance for carbohydrate metabolism is well-known, its contribution to the production of ribose-5-phosphate, which is substrate for DNA synthesis, suggests that WSSV may use the upregulation of metabolic genes such as G6PDH to provide essential substrate for viral replication (Figure 5).

G6PDH is also important to complete the development of *P. vindemiae* wasp, as the larvae can use the host hemolymphic carbohydrates as substrate to supply energy for development (Yang et al., 2020). The wasp enzyme causes transcriptional inhibition of *D. melanogaster* G6PDH, leading to increased G6P levels in the host, which can be used as a substrate for wasp development. In RNAi-treated wasps, a lower infectivity capacity is observed when compared to control insects, supporting the idea of immunometabolic interactions involving PPP enzymes (Yang et al., 2022). The findings discussed thus far highlight the promising potential of targeting the immune system and metabolic pathways, particularly PPP, for the development of novel strategies to control arthropods.

## 5 Conclusion

The precise regulation of G6PDH activity is of utmost importance for animals to adapt to diverse physiological and pathological conditions. While the significance of G6PDH has been extensively demonstrated in numerous organisms, we focus on its role in arthropods, particularly related to oxidative homeostasis, glucose metabolism and immunometabolic interactions. This underscores the critical involvement of G6PDH in various stages of arthropod metabolism, where it performs diverse physiological functions under different circumstances. It is worth noting that the research on G6PDH in arthropods is relatively limited compared to mammals. Therefore, the objective of this review is to pinpoint the importance of studying G6PDH in arthropods, bridging the existing gap in the literature.

In conclusion, by combining the understanding of glucose metabolism in vertebrates with experimental investigations in arthropods, we gain valuable insights into the multifaceted roles of G6PDH. Further exploration of energy metabolism in arthropods will advance our understanding of the PPP control mechanisms, contributing to an improved knowledge of their physiology. We believe that the particularities of PPP, and especially G6PDH, can be useful in developing new targets for arthropod control.
